# Bypassing the immunosuppressive effects of CA125/MUC16 via re-engineered rituximab (NAV-006) to improve its antitumor activity *in vivo*

**DOI:** 10.1093/abt/tbaf008

**Published:** 2025-04-24

**Authors:** Luigi Grasso, Bradford J Kline, Nicholas C Nicolaides

**Affiliations:** R&D Department, Navrogen Inc., Cheyney, PA 19319, United States; R&D Department, Navrogen Inc., Cheyney, PA 19319, United States; R&D Department, Navrogen Inc., Cheyney, PA 19319, United States

**Keywords:** ADCC, CDC, CD20, MUC16, CA125, rituximab, CD16

## Abstract

The monoclonal antibody rituximab functions through complement-dependent cytotoxicity (CDC) and antibody-dependent cellular cytotoxicity (ADCC) and is used to treat non-Hodgkin’s lymphoma. Elevated serum CA125/MUC16 levels, present in some follicular lymphoma patients, have been shown to correlate with reduced efficacy of rituximab. Previous studies revealed that CA125/MUC16 binds to rituximab, diminishing its CDC and ADCC. A rituximab variant, NAV-006, was engineered to counteract CA125/MUC16’s immunosuppressive effects. NAV-006 demonstrated enhanced CDC and ADCC activities and was unaffected by CA125/MUC16. In the present study, NAV-006 showed improved *in vivo* antitumor activity compared to rituximab in a human lymphoma model with reconstituted CA125/MUC16. Additionally, CA125/MUC16 bound to newer antibody-based lymphoma treatment agents, including obinutuzumab and tafasitamab, suppressing their immune effector functions. Bispecific antibodies mosunetuzumab and glofitamab also exhibited reduced cytotoxicity in the presence of CA125/MUC16. These findings suggest that NAV-006 could improve therapeutic efficacy in B-cell lymphomas, particularly in patients with elevated CA125/MUC16 levels.

## Teaser

Statement of Significance: Multiple recent studies have highlighted the immunosuppressive impact of MUC16/CA125 on antibodies immune effector function including rituximab (RITUXAN®). The present study presents data related to the engineering of a variant of rituximab that can escape MUC16/CA125 immunosuppressive effects resulting in improved efficacy *in vivo*.

## Introduction

Follicular lymphoma (FL) constitutes 20% of all non-Hodgkin’s lymphoma (NHL) cases, with an annual incidence of 2.5 new cases per 100,000 men and women in the USA [1]. Treatment for FL typically involves multiple therapy lines that often result in progressively shorter disease-free survival. Targeting CD20 on FL cells is a pivotal therapeutic approach that encompasses rituximab, a monoclonal anti-CD20 antibody used alone or in combination with chemotherapy. Rituximab works through complement-dependent cytotoxicity (CDC) [2] and antibody-dependent cellular cytotoxicity (ADCC) [3, 4]—collectively known as “effector function” mechanism. Several studies have identified CA125/MUC16 as a tumor microenvironment factor, often elevated (above the normal 35 U/ml level) in the serum of up to 40% of FL and other NHL patients [5]. While prevalent in FL and other cancers, we have only recently begun elucidating the role of CA125/MUC16 in tumor biology and immunity. High serum CA125/MUC16 levels have been shown to diminish the effectiveness of experimental therapeutic antibodies like farletuzumab and amatuximab [6–8], which, similar to rituximab, elicit immune effector function [9–11]. Given that ADCC and CDC are crucial to rituximab’s mode of action and CA125/MUC16 suppresses immune effector function, the correlation between elevated serum CA125/MUC16 levels and reduced responses to rituximab in FL patients is significant. In fact, rituximab demonstrated superior 5-year progression-free survival in FL patients with CA125/MUC16 levels <35 U/ml compared to those with CA125/MUC16 levels >35 U/ml [12]. This finding suggests that, akin to farletuzumab and amatuximab, immunosuppression by CA125/MUC16 may also hinder rituximab’s clinical efficacy. Recently, we demonstrated that CA125/MUC16 reduced rituximab-induced ADCC activity by inhibiting its engagement with the Fc-gamma receptor FCGR3A/CD16a (herein referred to as CD16) on effector cells [13]. We therefore engineered a novel rituximab variant named NAV-006 by using Block-Removed Immunoglobulin Technology (BRITE). NAV-006 proved resistant to CA125/MUC16’s immunosuppressive effects *in vitro*, showing enhanced CDC and ADCC activities [14]. Herein, we explore NAV-006’s *in vivo* antitumor efficacy and compare its *in vitro* performance against other non-rituximab anti-CD19 or CD20 agents currently under development for the treatment of B-cell lymphomas.

## Materials and methods

### CA125/MUC16 binding and ADCC

CA125/MUC16 binding and ADCC assays were carried out as previously reported [14]. CA125/MUC16 was purchased from Lee Biosolutions (purity ≥ 95% according to vendor’s certificate of analysis). The percentage of ADCC suppression was calculated by the formula:


\begin{align*}&\left( \frac{\text{cell viability with CA125}}{\text{cell viability without CA125}} - 1 \right) \times 100\end{align*}

### Animals

Immunodeficient NCG female mice (Charles River Laboratories), age 6 to 10 weeks, were maintained in individually ventilated cages in a dedicated pathogen-free environment. Animal experiments were approved by the Institutional Animal Care and Use Committee. Efforts were made to minimize animal suffering, and animal health was monitored by licensed veterinarians.

### Human peripheral blood mononuclear cells

Peripheral blood mononuclear cells (PBMCs) isolated from healthy donors used for ADCC and *in vivo* studies were purchased from BioIVT (Santa Clara, CA). Subject identifiers linked to the specimens were not available, therefore our studies are considered exempt from Federal regulations for the Protection of Human Subjects. For *in vivo* implantation, each vial of cryopreserved PBMCs was thawed and cells were washed using complete RPMI supplemented with fetal bovine serum (FBS, 10%). Cells were resuspended in 5 ml complete RPMI +10 ng/ml human IL-2 and incubated at 37 °C in 5% CO_2._ The following day, viable PBMCs were counted and resuspended in PBS + 2% FBS and immediately injected intraperitoneally (i.p) into mice.

### Human lymphoma model

NCG mice were implanted i.p. with human lymphoma Raji cells (200 000/mouse) expressing the reporter firefly luciferase. Mice were reconstituted with a single i.p. injection of human CA125/MUC16 (58 000 units/mouse) as well as with human PBMCs (4 million/mouse) prepared as described. A single i.p. dose of either rituximab or NAV-006 antibody treatment (1 mg/kg) was given i.p. with the tumor cells implantation. For bioluminescence imaging, D-Luciferin substrate was administered i.p. at 150 mg/kg. Ventral images of anesthetized animals were taken 10 minutes post-substrate injection on Days 14 and 28 after the start of antibody treatment. Study termination was planned on Day 28.

### Immunohistochemistry

To confirm engraftment of human PBMCs in mice, we employed immunohistochemistry (IHC) using a rabbit anti-human CD4 antibody (10400-R113, Sino Biological). Briefly, 5 µm sections of formalin-fixed, paraffin-embedded spleens were adhered to glass slides, deparaffinized and prepared for antigen retrieval in boiling 10 mM sodium citrate pH 6 solution for 10 minutes. Tissues were then equilibrated with phosphate-buffered saline-0.05% Tween-20 (PBS-T) and quenched for endogenous peroxidase activity using a 0.3% peroxidase/methanol solution for 10 minutes and then blocked for 1 hour in 10% goat serum in PBS-T. Sections were rinsed in PBS-T and probed for 1.5 hours with anti-human CD4 antibody (diluted in blocking buffer to 3 µg/ml), followed by PBS-T washing. Subsequently, slides were probed with 5 µg/ml of anti-rabbit, HRP-conjugated secondary antibody for 1 hour. Control slides were incubated with no primary antibody. Slides were then washed and brown staining was developed using eBioscience™ DAB advanced chromogenic substrate as recommended by the manufacturer (ThermoFisher). Slides were counterstained with hematoxylin, cover-slipped and analyzed for antigen expression under light microscopy using a Zeiss Axioplan wide-field microscope.

### Statistics

*P* values for data generated by enzyme-linked immunosorbent assay (ELISA) and *in vitro* cell killing or activation assays were calculated using the Student’s *t*-test. All other *P* values were calculated via one- or two-way Analysis of Variance (ANOVA), with Dunnett’s or Tukey’s multiple comparisons test using Prism 10.2 software. Results were considered to be significant if *P* < 0.05.

## Results

### *In vitro* performance of various antibody formats in the presence of CA125/MUC16

**Figure 1:**
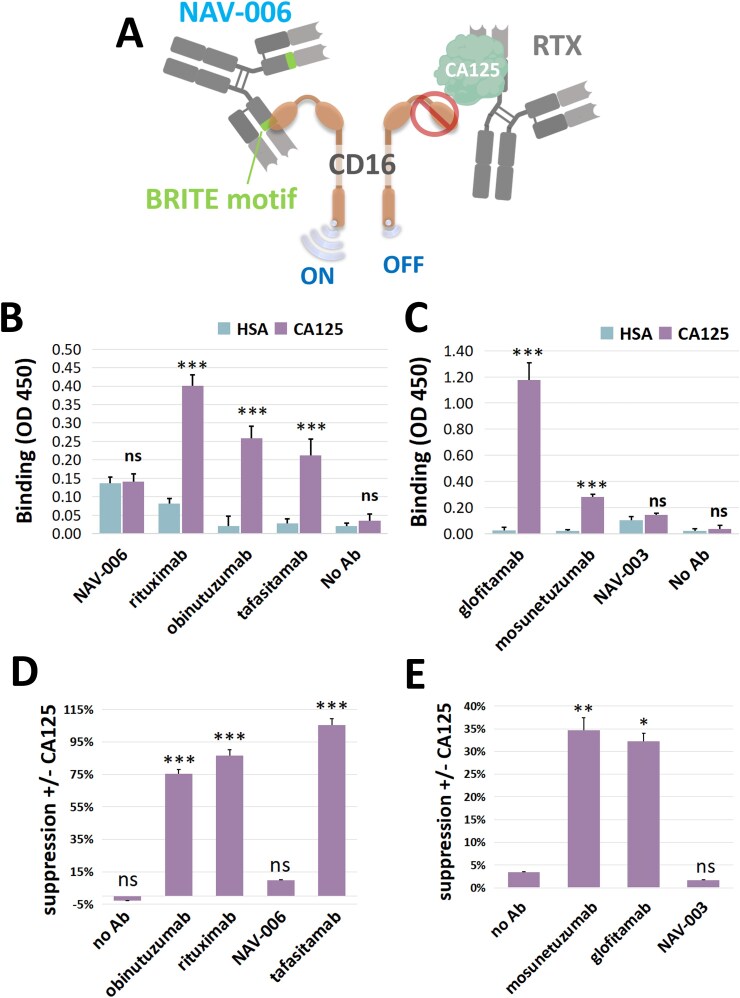
(A) Schematic of NAV-006 mechanism of action: The BRITE motif engineered in NAV-006 enables it to evade CA125/MUC16 blockade and activate the CD16 receptor, enhancing its effector function (ADCC/CDC). CD16 signaling activation (“on”) or inhibition (“off”) are also depicted. (B) Similar to rituximab, CA125/MUC16 bound to both obinutuzumab and tafasitamab while CA125/MUC16 binding to NAV-006 was minimal. In all experiments, human serum albumin (HSA) was used as a non-specific binding control protein. (C) CA125/MUC16 bound to both the bispecific agents mosunetuzumab and glofitamab but not to NAV-003, an MSLN-directed CD3 bispecific agent previously reported to have minimal CA125/MUC16 binding. Also shown is the percent of immunosuppression mediated by CA125/MUC16 on the cytotoxicity mediated by IgG-based obinutuzumab and tafasitamab (D), and CD20/CD3 bispecific mosunetuzumab and glofitamab (E). NAV-003 was included as a negative control. A representative experiment from two independent experiments is shown. All data represent biological replicates and are expressed as mean ± standard deviation. An unpaired *t*-test was used for comparison between experimental groups; ns = not statistically significant; ^*^ = *P* < 0.05; ^**^ = *P* < 0.01; ^***^ = *P* < 0.001.

We have previously reported that CA125/MUC16 binds to rituximab and reduces its interaction with CD16 as schematically shown in Fig. 1A (right-hand side). CA125/MUC16 binding interface within the IgG appears to span across both its heavy chain framework 4 and complementarity determining region 3 and its CH1 domain [14]. CA125/MUC16 binding leads to allosteric changes in the CH2 domain of the Fc region where the canonical binding sites for both C1q and Fc gamma receptor are located [14]. This interaction results in reduced CD16 signaling and suppressed ADCC as well as CDC [13, 14]. In the rituximab variant NAV-006, a single amino acid change (BRITE motif) in its variable region [14] significantly reduced CA125/MUC16 binding and enhanced CD16 binding and signaling, and ultimately effector function (Fig. 1A, left-hand side). This amino acid change does not affect NAV-006’s binding to CD20 [14]. Despite some patients developing resistance, rituximab remains the preferred treatment due to its long-standing safety record and lower costs compared to newer anti-CD20 antibodies like obinutuzumab, which has shown promise but is associated with higher adverse event rates and higher costs [15]. Tafasitamab is an FDA-approved humanized monoclonal antibody targeting the pan B-cell antigen CD19 and used in the treatment of relapsed/refractory (R/R) diffuse large B-cell lymphoma (DLBCL) [16]. Fc modification in tafasitamab increases binding affinity to Fc receptors on effector cells, enhancing ADCC. We sought to determine whether CA125/MUC16 may impact the activity of these newer antibody-based strategies. Similar to rituximab, CA125/MUC16 bound to both obinutuzumab and tafasitamab (Fig. 1B), while CA125/MUC16 binding to NAV-006 was minimal. In all experiments, human serum albumin was used as a non-specific binding control protein. Furthermore, the interaction of CA125/MUC16 with obinutuzumab and tafasitamab was accompanied by a substantial reduction in their effector functions in the presence of CA125/MUC16 (Fig. 1D); by contrast, NAV-006 was unaffected as compared to rituximab as previously reported.

**Figure 2:**
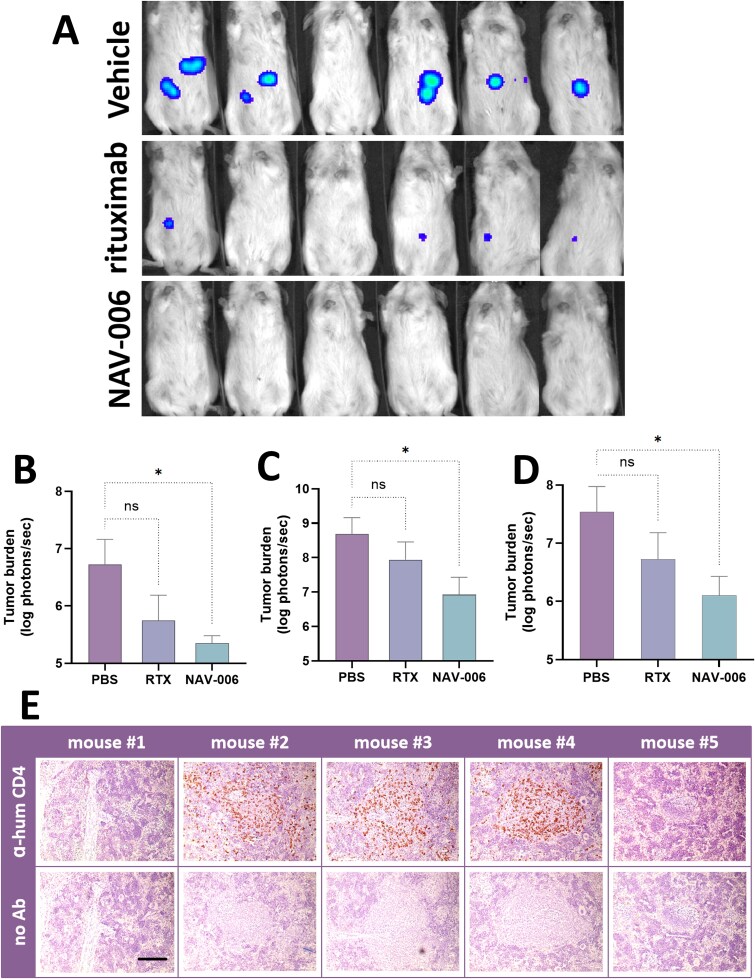
(A) Representative whole-body, ventral bioluminescence imaging of mice implanted with human lymphoma Raji cells expressing the reporter firefly luciferase. On day 14 after the start of treatment, tumor lesions in NAV-006-treated mice (bottom panel) are undetectable, whereas they are visible in vehicle-treated mice (top panel). Smaller lesions are detectable in some of the mice treated with rituximab (center panel). (B-C) independent studies using the same model as in (A); in both examples, the results show statistically significant antitumor activity mediated by NAV-006, while rituximab effect fails to achieve statistical significance. (D) Data from studies in (B) and (C) were pooled and statistical analysis is presented in a combined chart. One-way ANOVA with Dunnett’s multiple comparison test; ns = not statistically significant; ^*^ = *P* < 0.05. (E) Immunohistochemistry analysis for detection of CD4-positive human lymphocytes. Spleen tissues harvested from five representative mice 28 days after the start of treatment were probed with an anti-human CD4 antibody as described in materials and methods. Three of five animals had spleens colonized by human lymphocytes even 4 weeks after PBMCs transfer. Scale bar: 200 µm.

Another antibody-based format being developed for the treatment of B-cell lymphoma includes bispecific antibodies [17]. In recent years, the Food and Drug Administration granted approval to both mosunetuzumab (Lunsumio™), a first-in-class CD20-directed CD3 T-cell engager for adult patients with R/R follicular lymphoma, as well as glofitamab (Columvi™), a CD20-directed CD3 bispecific for the treatment of R/R DLBCL. Because bispecific antibodies have a different mode of action than rituximab, we sought to determine whether CA125/MUC16 may also impact their activity. CA125/MUC16 could bind to both mosunetuzumab and glofitamab but not to NAV-003 (Fig. 1C and Fig. S1). NAV-003 is an MSLN-directed CD3 bispecific agent that we previously reported to have minimal CA125/MUC16 binding [18]. In addition, we observed significant suppression of mosunetuzumab and glofitamab cytotoxicity in the presence of CA125/MUC16 (Fig. 1E).

### NAV-006 antitumor activity against human lymphoma

We have previously demonstrated that the *in vitro* tumor cell killing activity by NAV-006 is improved compared to parent rituximab due to its reduced binding to the immunosuppressive factor CA125/MUC16 [14], as also shown in Fig. 1. It was thus important to test whether NAV-006 could mediate anti-tumor activity *in vivo* that is comparable and potentially improved compared to rituximab in the presence of human CA125/MUC16. As there are no animal models that can mirror the high levels of CA125/MUC16 as found in certain human tumors, CA125/MUC16 needed to be reconstituted in the mice used for efficacy studies. Additionally, to recreate the human immunological condition as found in patients, human PBMCs were transferred into immunodeficient mice. Human lymphoma was reconstituted by cell-transfer of CD20-positive Raji cells originally isolated from a lymphoma patient [19]. Tumor burden was monitored by luminescence imaging (Fig. 2A) and specific details of this model are described in Materials and Methods. Under these conditions, rituximab showed meaningful antitumor activity; however, its effect was not statistically significant. In contrast, NAV-006 showed improved efficacy that met statistical significance (Fig. 2B-D). On average across experiments, tumor growth inhibition by rituximab and NAV-006 was 73% and 95%, respectively, with a statistically significant difference between the means of the groups (*P* ≤ 0.033) as tested by one-way ANOVA. Furthermore, immunohistochemistry analysis determined that most of the mice retained a substantial population of human CD4-positive cells in lymphoid tissues, such as in the spleen, even after 4 weeks post PBMCs transfer, indicating successful human effector cells engraftment (Fig. 2E). These results support the hypothesis that NAV-006, as a single agent, could be more beneficial than rituximab in clinical situations where CA125/MUC16 immunosuppression may reduce the treatment-induced response rate.

## Discussion

Here, we show that the immunosuppressive effects mediated by CA125/MUC16 are not limited to rituximab but also extend to newer agents that utilize either a monospecific IgG or a bispecific format. CA125/MUC16 binds to the bispecific antibodies mosunetuzumab and glofitamab, suppressing their cytotoxicity and indicating that CA125/MUC16 can also impact their effectiveness. We plan to further investigate the molecular mechanism by which CA125/MUC16 mediates these effects upon binding these bispecific agents. Our current hypothesis is that the interaction of CA125/MUC16 with these agents causes a change in their interdomain spacing and/or spatial configuration, as previously described [20]. In addition, due to its massive molecular size (1000+ kDa), CA125/MUC16 may hinder and thus reduce CD3 engagement.

NAV-006, minimally affected by the presence of CA125/MUC16, maintains its effector function, suggesting its potential superiority over rituximab in overcoming CA125/MUC16-mediated immunosuppression. In a reconstituted human lymphoma mouse model, NAV-006 demonstrated significantly improved anti-tumor activity compared to rituximab in the presence of human CA125/MUC16. We plan to conduct further testing of NAV-006 in additional models to expand its potential clinical utility. It would also be beneficial to study its activity in combination with the chemotherapeutic agents currently used with rituximab, as well as to explore alternative dosing regimens.

## Supplementary Material

supplementary_fig_1_01anther_tbaf008

## Data Availability

The data that support the findings of this study are available from the corresponding author upon reasonable request.

## Animal research statement

Animal experiments were approved by the Animal Care and Use Committee at Charles River Laboratories. Efforts were made to minimize animal suffering and animal health was monitored by licensed veterinarians.
