# Supplementary figures and images for: Bypassing the immunosuppressive effects of CA125/MUC16 via re-engineered rituximab (NAV-006) to improve its antitumor activity in vivo
Source: Antib Ther. 2025 Apr 24;8(3):171–6. doi: 10.1093/abt/tbaf008 (PMC12199351; doi:10.1093/abt/tbaf008)

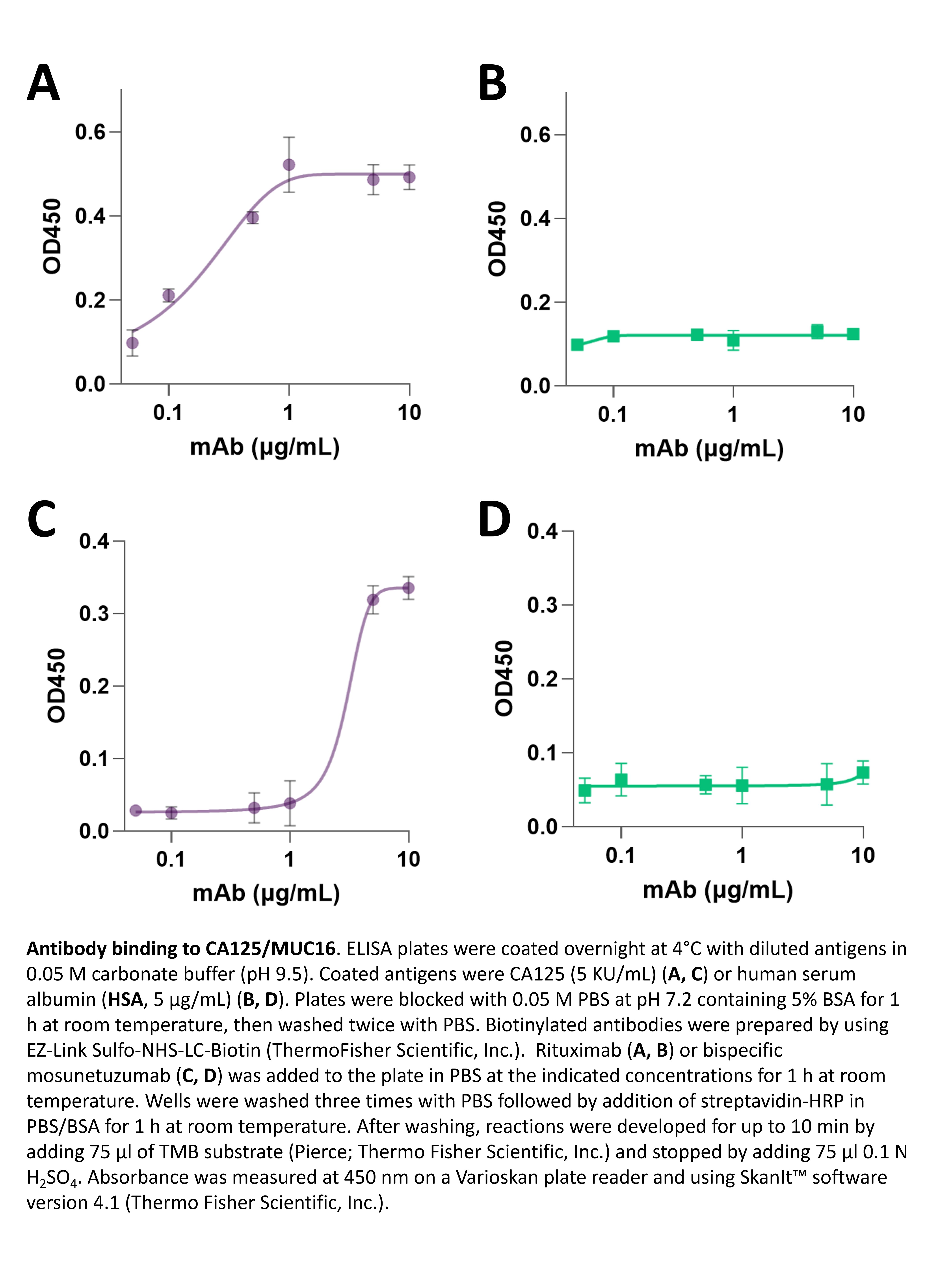

Supplement: supplementary_fig_1_01anther_tbaf008 [file supplementary_fig_1_01anther_tbaf008.jpeg]
